# Book Review: R. Dingwall and S. Frost (eds.), Health and Safety in a Changing World, Routledge, London, 2017: Evolving Goals for Health and Safety in Workplaces Today

**Published:** 2017-07-01

**Authors:** G. Cersosimo

**Affiliations:** 1 Department of Medicine, Surgery and Dentistry – Scuola Medica Salernitana -University of Salerno (Italy)

Collective action, the result of analyses and reflections by intellectuals, health professionals, union officials, public institutions and private foundations, has historically helped develop health and safety practices in the workplace, by modifying and/or progressively strengthening the concept of Health For All (Porter, 1999).

One main reference point in this process of transformation, is the progressive and constant attention paid to the labor sector with emphasis placed on two pivotal components: 1) workers (women, men and too often children) and 2) workplaces (space, conditions and hours worked) in relation to specific harmful aspects.

This book is the result of a ten-year collaboration supported by the Institution of Occupation, Safety and Health (IOSH) and based on research modeled on the global and changing, professional goals and standards for health and safety.

All six contributions give voice to the re-determination of health and safety as intertwined elements linked to the “*increasing complexity of the organizational environment*” where professions and related tasks are developed and where working conditions may erode workers.

It is true in the United Kingdom as elsewhere that “health and safety stakeholders and practitioners” are not the uniquely involved subjects [2] but rather, all individuals who are aware of the importance of personal health and safety in society play a role.

The curators of this book (Dingwall and Frost) express similar fundamental ideas in the Introduction: “*that every worker should finish his work alive, in good health …and with same number of organs that he started with*”. This assertion is restated in the last pages of the book as well, and represents both a goal for a long-lasting project of protection embedded within welfare, health and safety policies, and a reflection on the historical connection between health and work.

The reference to “the number of organs” is sadly coherent with the remarks of Karen McDonnell and Bill Gunnyeon (IOSH) in the preface where they consider the seemingly hidden yet massive global deregulation of workplaces: “*more than two million people die every year as result of health and safety failures at their workplace*”. Furthermore, this number (two million) does not account for the toll of elusive and undocumented workers whose names are not on record, yet whose lives are sacrificed in the workplace everyday.

Most of the research found here underlines a need for job safety in big and small businesses alike. One of the most significant barriers to ensuring job safety is the ever-changing nature of the work environment in factories and organizations. To this end, awareness is key when extending controls and safety measures.

The editors and authors underline that IOSH responsibility and engagement have been modified adequately during the last 20-year period of transformation. They are also aware that in an international context, these regulations should be based on a flexible working model, one that continues to adjust the rules, networks, instruments and production techniques to specific technological infrastructures as a foundation and support structure.

It should be recognized that these are ongoing and unresolved questions, historically linked to the difficulty of improving workplaces in a time of rapid transformation in which analyses and results quickly become obsolete. Further, these questions must be considered in relation to the needs and desires of various actors, intellectual and technical operators, as well as large and small organizations.

The previous modes of ensuring *Health for All* are lacking in relation to workers and working conditions today. What we need collectively, are new approaches that will foster awareness of the importance of health and safety in workplaces around the world, and a new perspective in relation to current professions and professionals.

The innovative and unconventional ideas presented here are a reflection of the current, globalized nature of business. As we move further in this direction, it is important to maintain an equally open and global perspective in relation to the ever-evolving goals of health and safety in workplaces today.

## References

[1] Porter D (1999). Health, Civilization and the State A History of Public Health from Ancient to Modern Time.

[2] Leka, Dingwall R, Frost S (2017). Health and Safety in a Changing World.

